# The Role of Surgery in High-Grade Neuroendocrine Cancer: Indications for Clinical Practice

**DOI:** 10.3389/fmed.2022.869320

**Published:** 2022-03-25

**Authors:** Francesco Petrella, Claudia Bardoni, Monica Casiraghi, Lorenzo Spaggiari

**Affiliations:** ^1^Department of Thoracic Surgery, IRCCS European Institute of Oncology, Milan, Italy; ^2^Department of Oncology and Hemato-Oncology, University of Milan, Milan, Italy

**Keywords:** surgery, small cell lung cancer, large cell neuroendocrine carcinoma, lobectomy, pneumonectomy

## Abstract

Pulmonary neuroendocrine tumors (pNET) represent a particular type of malignant lung cancers and can be divided into well-differentiated low-grade NET and poorly-differentiated high-grade NET. Typical and atypical carcinoids belong to the first group while large cell neuroendocrine carcinomas (LCNEC) and small-cell lung cancers (SCLC) belong to the second one. The aim of this mini-review is to focus on the role of surgical therapy for high grade neuroendocrine tumors. SCLC has the worst prognosis among all lung cancer neoplasms: in fact, the two-year survival rate is about 5% and median survival usually ranges between 15 and 20 months. The surgical treatment of SCLC has thus infrequently been judged as a valuable aspect of the therapeutic approach, the gold standard treatment being a combination of platinum-based chemotherapy and radiotherapy. As LCNEC are rare, there is a lack of extensive literature and randomized clinical trials, therefore the curative approach is still controversial. Current treatment guidelines suggest treating LCNEC by surgical resection in non-metastatic stages and recommend adjuvant chemotherapy according to SCLC protocol. Upfront surgery is suggested in early stages (from I to IIB), a multimodality approach is recommended in locally advanced stages (III) while surgery is not recommended in stage IV LCNEC. The rate of surgical resection is quite low, particularly for SCLC, ranging from 1 to 6% in limited diseases; lobectomy with radical lymphadenectomy is considered the gold standard surgical procedure in the case of limited disease SCLC and resectable LCNEC; pneumonectomy, although reported as an effective tool, should be avoided in the light of local and distant recurrence rates.

## Introduction

Pulmonary neuroendocrine tumors (pNET) represent a particular type of malignant lung cancers and can be divided into well-differentiated low-grade NET and poorly-differentiated high-grade NET. Typical and atypical carcinoids belong to the first group while large cell neuroendocrine carcinomas (LCNEC) and small-cell lung cancers (SCLC) belong to the second one (1).

High grade neuroendocrine tumors present significantly higher mitotic rates when compared to low-grade neuroendocrine tumors; moreover, increased necrosis is commonly observed as well as their combination with other types of lung cancer like adenocarcinomas or squamous cell carcinomas (2).

The vast majority of high-grade neuroendocrine tumor patients are older and heavy smokers, with an early tendency to metastasize and a globally poor long-term prognosis with 5-year survival rates ranging from 15 to 57% (3–5).

SCLC account for 15–20% of all pulmonary tumors; among them, only 10 – 20% of cases are early-stage tumors amenable to curative local treatments; on the contrary, the vast majority of patients present huge and centrally-located lesions – very often causing superior vena cava compression and/or infiltration - and early dissemination, chemotherapy thus being the most effective fist-line treatment (6, 7). About 10% of patients suffering from SCLC present paraneoplastic syndromes such as Lambert-Eaton Syndrome, Cushing syndrome, hypercalcemia and syndrome of inappropriate antidiuretic hormone secretion (SIADH) (7).

LCNEC account for <1% of all lung cancers and 40% of affected patients are diagnosed in metastatic stage (8). Histological differential diagnosis between SCLC and LCNEC can be difficult because of the many common features shared by the two diseases, such as necrosis, neuroendocrine morphology, positive immunohistochemical staining for neuroendocrine markers and a high mitotic rate (9) (Table 1).

**Table 1:** Literature review.

**SCLC**		
	Fox et al. (10) Lad et al. (11)	No survival benefit of neoplasm resection.
	HwihdJiang et al. (12) Lim et al. (13) Tsuchiya et al. (14)	Resected SCLC disclosed a median survival of 20 months and a 5-year survival of 11.1–52%.
	Schreiber et al. (15)	Resected T1-2 SCLC disclosed a median overall survival of 42 vs. 15 months; T3-4 22 vs. 12 months.
	Casiraghi et al. (16)	1, 5 and 10-year overall survival rates of 73.6%, 42% and 25.6%.
**LCNEC**		
	Iyoda et al. (17)	5-year survival rate of resected LCNEC after induction treatment is reported to be 88%, while without induction treatment it falls to 47%.
	Veronesi et al. (4)	LCNEC with mediastinal lymph node metastases show a significantly worse prognosis.
	Girelli et al. (18)	Mediastinal involvement had a significantly worse prognosis when compared to pN0 patients.
	Lo Russoet al. (19)	Radical resection should always be attempted whenever feasible and patients with nodal involvement should always receive adjuvant treatments.

## The Role of Surgery in Small Cell Lung Cancer

Small cell lung cancer (SCLC) has the worst prognosis among all lung cancer neoplasms: in fact, the two-year survival rate is about 5% and median survival usually ranges between fifteen and 20 months (7). It is characterized by early and fast diffusion, presenting a significant recurrence rate after the initial response to treatments (20). The surgical treatment of SCLC has thus infrequently been judged as a valuable aspect of the therapeutic approach, the gold standard treatment being a combination of platinum-based chemotherapy and radiotherapy (20, 21).

Two randomized controlled trials performed in the 70's and 90's evaluated the contribution of surgical resection to the therapeutic pathway of limited disease-small cell lung cancer: although some important limitations of both studies emerged, none of them was able to find any survival benefit of neoplasm resection (10, 11). In more recent times, small series of surgical resection of SCLC - focusing on different outcomes - have been reported, disclosing a median survival of 20 months and a 5-year survival of 11.1–52% (12–14). Nowadays, operated limited disease – small cell lung cancer represents only a small percentage of resected lung tumors, accounting for 0–6.1% of all resected pulmonary neoplasms (22), although several large prospective cohort studies have recently shown a potential benefit of operating early stage SCLC (15, 22–24). T1 and T2 SCLC resected diseases disclosed a median overall survival benefit of 42 vs. 15 months as well as T3 and T4 disease (22 vs. 12 months) (15). Sub-lobar resections are not suggested, as they show a significantly worse prognosis when compared to anatomical resection (24).

Locally advanced SCLC (stage IIIa) should not be considered for surgery as suggested in almost no guidelines (25–29); nonetheless, radical lymphadenectomy in N2 patients has been reported to have a valuable impact on survival in several series (15, 23, 30).

In our personal retrospective experience, we observed 65 patients suffering from SCLC and surgically treated with curative intent. Our results disclosed a median overall survival of 36 months and postoperative 1, 5 and 10-year overall survival rates of 73.6, 42, and 25.6%. In particular, patients receiving surgical radical resection and presenting a pathological stage I had a 5-year overall survival of 76.6%; on the contrary, patients undergoing induction treatments or adjuvant radiotherapy had a worse prognosis probably due to a more advanced stage with lymph node involvement. In fact, lymph node involvement together with volume and site of the tumor were significantly related to overall survival, pT4 or pN2 patients presenting 1-year overall survival rates of 50 and 57.1% respectively; none of them was alive at 5 years (16).

The role that surgery may play for treating limited-stage SCLC remains unclear due to controversial literature results and the absence of recent randomized clinical trials.

Considering how easily SCLC tends to metastasize and its high chemo-sensitivity, many recent guidelines suggest a non-surgical approach to limited disease-SCLC, recommending platinum-based chemotherapy and mediastinal radiotherapy, or chemotherapy alone with prophylactic cranial irradiation for more advanced diseases (31, 32).

Recent larger retrospective series have shown possible advantages offered by the resection of limited-stage SCLC. Encouraging 5-years overall survival rates of 48, 39, and 15% for operated patients in stage I, II and III respectively have been shown by The International Association for the Study of Lung Cancer (IASLC) Lung Cancer Staging Project in a group of 349 patients (23).Similarly, the Surveillance, Epidemiology and End Results study (SEER) disclosed a 5-year overall survival rate of 50.3% in a retrospective series of 247 resected stage I SCLC patients (33), and Yang et al. reported a 5-year survival rate of 47% in a cohort of 1,574 early stage-SCLC patients from the National Cancer Database, receiving radical resection (34).

The role of surgery in early stage SCLC might be not only limited to an improvement of overall survival but also to an appropriate histo-pathological diagnosis, thus supporting SCLC histology which could be misdiagnosed in the case of mixed forms, some NSCLC or rare tumors, thus adapting the treatment plan to the new acquired histology and helping the formulation of a different prognosis (35, 36).

To date, there is no evidence supporting surgical indication in stage II and stage IIIA SCLC. NCCN guidelines, in fact, do not recommend resecting advanced tumors as they do not benefit from surgery (35), although some recent reports seem to disclose a significant improvement in survival in stage II and stage IIIA SCLC undergoing lung resection (37). Nevertheless, whenever a surgical option is offered to SCLC patients, a careful balance between expected benefits and risks should be carried out, taking into consideration the volume extension of the planned resection, the clinical stage of the disease and the performance status of the patient; a multidisciplinary discussion is strongly recommended and every available less invasive therapeutic option should be contemplated (38).

The surgical approach to limited disease-SCLC should be standard lobectomy with lymphadenectomy which provides the best overall survival, in particular when compared to lesser resection such as wedge resection (39); on the other hand, the role of pneumonectomy in SCLC is unclear and, taking into consideration the disease's biology and the high risk postoperative course, it should be avoided even in salvage settings (40, 41).

A more effective role of surgery has been observed within a multimodality approach including chemotherapy and/or radiotherapy in patients presenting a resectable disease (42). The NCCN guidelines, in fact, recommend adjuvant chemotherapy even in the case of N0 disease at clinical staging; moreover, they suggest sequential or concurrent chemo and radiotherapy in N+ disease, reporting a more effective role of radiotherapy in pN2 disease than in isolated N1 disease (43, 44).

Worth of being reported is a combined form of SCLC and NSCLC which is relatively rare and it is defined as SCLC combined with any elements of non-small cell lung cancer (45). Incidence of combined SCLC has been reported to range from 2 to 28% and its prognosis does not significantly differ from pure SCLC after surgical resection (45).

## The Role of Surgery in Large Cell Neuroendocrine Carcinomas

It has been widely demonstrated that is quite difficult to obtain a precise diagnosis before surgery in the case of LCNEC; in the vast majority of cases, in fact, a definitive pathological confirmation is acquired by analyzing resected specimens (46–48). As LCNEC are rare, there is a lack of extensive literature and randomized clinical trials, therefore the curative approach is still controversial (49).

LCNEC shows a significantly worse prognosis when compared to other large cell non-neuroendocrine lung cancers (50, 51). A sex-related difference in terms of overall survival has occasionally been reported (52) but not further confirmed (53, 54). As for NSCLC, LCNEC with mediastinal lymph node metastases show a significantly worse prognosis (4).

Although the lack of randomized controlled trials and the retrospective nature of published studies do not allow definitive conclusions about the role of induction therapy or adjuvant treatments, it is quite well known that LCNEC is most often responsive to platinum-based neoadjuvant treatments (55). In fact, the 5-year survival rate of resected LCNEC after induction treatment is reported to be 88%, while without induction treatment it falls to 47% (17). Chemotherapy seems to play an additional beneficial role even in early stage LCNEC (4) although discordant results have been reported (56); nevertheless, taking into consideration the biological similarity of LCNEC to SCLC and the similar response rate, it seems reasonable to offer platin-based chemotherapy not only to advanced stage LCNC but also to early ones (17, 49). In our personal experience, patients with mediastinal involvement had a significantly worse prognosis when compared to pN0 patients (18); previous reports had already recommended aggressive combined approaches – as for SCLC –particularly in cases with lymph node involvement (57). Although in our experience no chemotherapy regimen conditioned overall survival, it has been widely reported that radical resection should always be attempted whenever feasible and patients with nodal involvement should always receive adjuvant treatments (19).

Current NCCN treatment guidelines suggest treating LCNEC by surgical resection in non-metastatic stages and recommend adjuvant chemotherapy according to SCLC protocol (44). Upfront surgery is suggested in early stages (from I to IIB), a multimodality approach is recommended in locally advanced stages (III) while surgery is not recommended in stage IV LCNEC.

## Conclusion

SCLC and LCNEC are high-grade neuroendocrine neoplasms; they grow faster than other NSCLC and show a more aggressive behavior and worse prognosis. While SCLC usually present as centrally-located bulky lesions, LCNEC are more frequently diagnosed as peripheral neoplasms. They are typically detected in heavy smoker older patients in stage IV at first diagnosis in 60–80% of cases in SCLC and 40% of cases in LCNEC. The rate of surgical resection is quite low, particularly for SCLC, ranging from 1 to 6% in limited diseases; lobectomy with radical lymphadenectomy is considered the gold standard surgical procedure in the case of limited disease SCLC and resectable LCNEC; pneumonectomy, although reported as an effective tool, should be avoided in the light of local and distant recurrence rates. The surgical route should always be evaluated within a multimodality approach including chemotherapy and radiotherapy in almost every stage.

## Author Contributions

FP, CB, MC, and LS took part in all the aspects of the paper, idealization, writing, and revision. All authors contributed to the article and approved the submitted version.

## Conflict of Interest

The authors declare that the research was conducted in the absence of any commercial or financial relationships that could be construed as a potential conflict of interest.

## Publisher's Note

All claims expressed in this article are solely those of the authors and do not necessarily represent those of their affiliated organizations, or those of the publisher, the editors and the reviewers. Any product that may be evaluated in this article, or claim that may be made by its manufacturer, is not guaranteed or endorsed by the publisher.
